# Acute onset of hip pain and swelling with calcification mimicking pyogenic myositis

**DOI:** 10.1002/ccr3.7142

**Published:** 2023-03-24

**Authors:** Kazuhiro Ishikawa

**Affiliations:** ^1^ Department of Infectious Diseases Tokyo Medical University Ibaraki Medical Center Ibaraki Japan

**Keywords:** calcific tendonitis, myositis of gluteus medius, pyomyositis

## Abstract

In cases with acute hip pain, noninflammatory orthopedics diseases are common. But we sometimes experience acute pyomyositis with high inflammation. Although a rare case, we consider calcific tendonitis and pyogenic myositis.

## 1 CASE PRESENTATION

The diagnosis of acute myositis with calcification is calcific tendonitis and pyogenic myositis. The evolution on NSAIDS is favorable for calcific tendonitis, but we need to administer antibiotics for the pyomyositis. We experienced the difficult case of the calcific tendonitis of the gluteus medius muscle.

**FIGURE 1:**
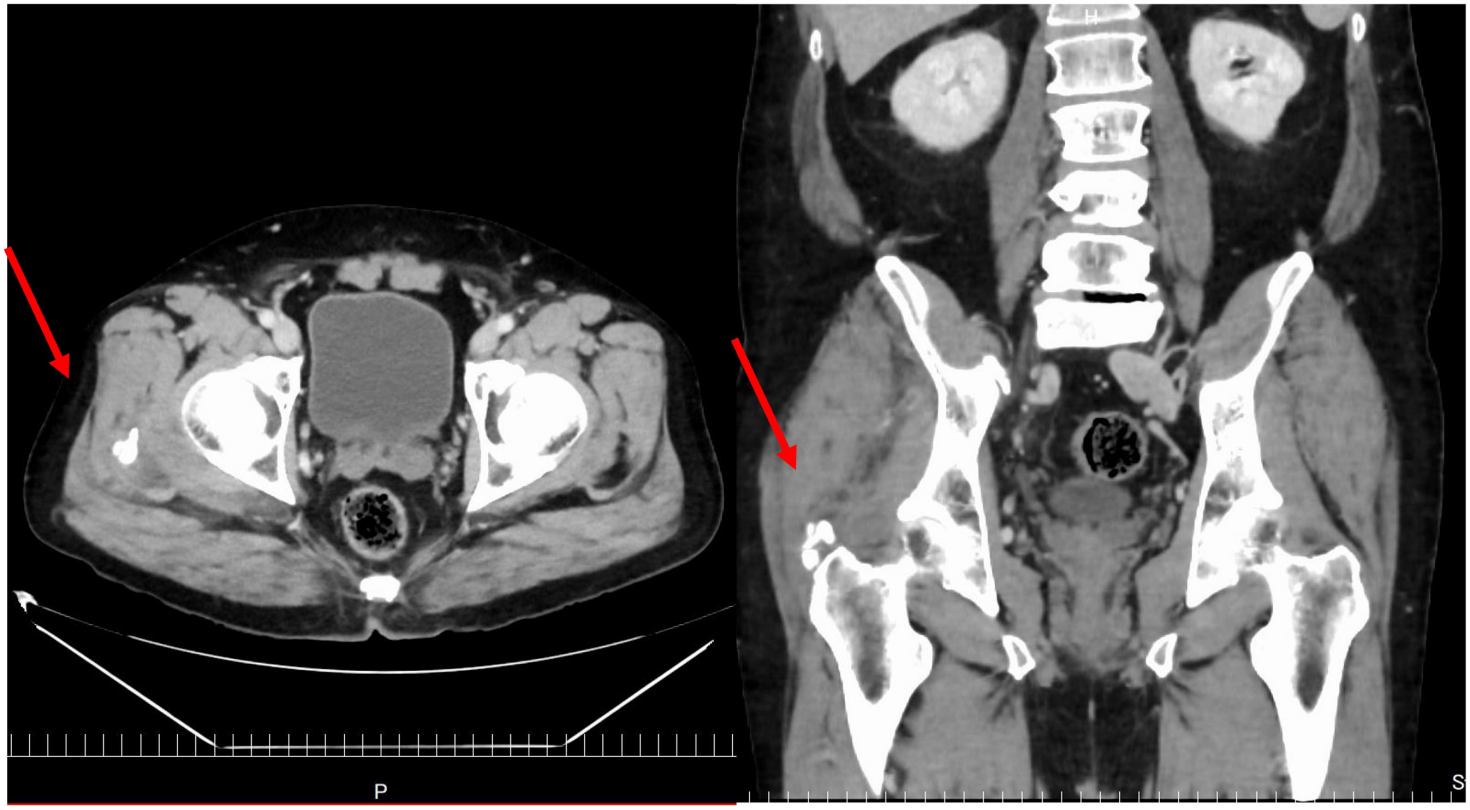
Left; axial: right; coronal; red arrow pointing at the calcification. Computed tomography scan with contrast showed a calcification (1.5 cm x 1.7 cm) slightly proximal to the right femoral tuberosity and edema of the surrounding soft tissues, including the gluteus medius muscle.

**FIGURE 2:**
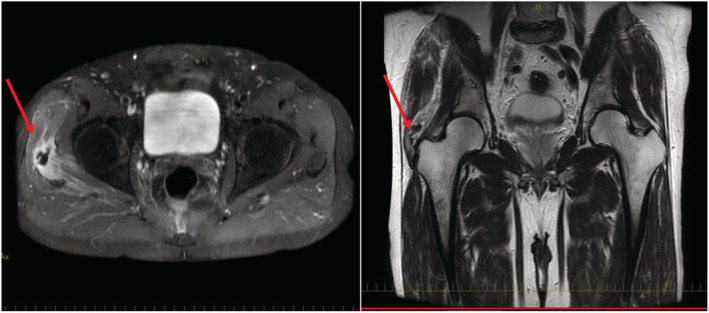
Magnetic resonance imaging revealed low‐intensity proximal to the greater tuberosity of the right femur on T1 and T2, and high intensity of muscles around the calcification on STIR and contrast enhancement.

A healthy 56‐year‐old man visited our hospital with acute onset of right hip pain and high fever. The patient had not been exposed to tuberculosis (TB). The symptoms persisted, and he experienced muscle weakness. He then consulted in our hospital. On admission, laboratory data revealed white blood cells at 7500/μL (neutrophil, 82.5%), a C‐reactive protein at 12.6 mg/dL, and T‐SPOT was negative. Chest radiograph was unremarkable. Contrast CT scan showed a calcification and edema of the gluteus medius muscle (Figure 1). We empirically started intravenous vancomycin plus cefazolin for suspected *Staphylococcus aureus* (*S. aureus*) infection. Blood culture was negative. MRI revealed high intensity of muscles around the calcification (Figure 2). We finally concluded in calcific tendonitis. We initiated treatment with nonsteroidal anti‐inflammatory drugs(NSAIDS) and stopped the antibiotics. His symptoms subsided, and his CRP improved to 5.4 mg/dL. The patient was discharged from the hospital. During follow‐up, he sometimes developed discomfort in the hip that improved on oral colchicine and NSAIDS.

## 2 DISCUSSION

Our differential diagnosis is calcific tendonitis and pyogenic myositis. Evolution on NSAIDS was favorable for calcific tendonitis. On the contrary, we need administer antibiotics for the pyomyositis. Calcific tendonitis is a common disorder of the shoulder of unknown etiology. Calcific deposits consist of poorly crystallized hydroxyapatite. Many cases resolve spontaneously or with conservative management. A very rare complication like our case is migration toward the myotendinous junction of the corresponding tendon, which causes a significant muscular inflammatory reaction leading to inflammation of the rectus femoris, piriformis, and iliopsoas muscles, and of the capsule. On the contrary, pyomyositis is a purulent infection of skeletal muscles that arise from hematogenous spread, usually with abscess formation. Predisposing factors to pyomyositis include immunodeficiency, trauma, and intramuscular drug use. *Staphylococcus aureus* is the most common cause of pyomyositis. There have also been cases of mycobacteria‐induced pyomyositis. TB causes soft tissue calcification^1^ of gradual onset, unlike the acute onset in the present case. In conclusion, we experienced noninfective myositis as a rare complications of calcified tendonitis.

## AUTHOR CONTRIBUTIONS

**Kazuhiro Ishikawa:** Conceptualization; data curation; formal analysis; investigation; methodology; project administration; supervision; validation; visualization; writing – original draft; writing – review and editing.

## FUNDING INFORMATION

This research was not supported by any specific grant from any funding agency in the public, commercial, or nonprofit sectors. Therefore, no funding body was involved in the design of the study, the collection, analysis, and interpretation of the data, the writing of the manuscript, or the decision to submit the manuscript for publication.

## CONFLICT OF INTEREST STATEMENT

The authors declare no conflict of interest.

## CONSENT

Written informed consent was obtained from the patient to publish this report in accordance with the journal's patient consent policy. All the authors express their consent for publication.

## Data Availability

Data available on request from the authors.
